# The human placenta releases substances that drive lung cancer into apoptosis

**DOI:** 10.1186/1746-1596-4-27

**Published:** 2009-08-21

**Authors:** Sebastian Marwitz, Tobias Zeiser, Holger Schultz, Daniel Kähler, Mahdi Abdullah, Hans-Peter Hauber, Peter Zabel, Ekkehard Vollmer, Torsten Goldmann

**Affiliations:** 1 Research Center Borstel, Clinical and Experimental Pathology, Parkallee 3a, D-23845, Borstel, Germany; 2 Paracelsus-Klinik, Section Gynecology, Wilstedter Str.134, D-24558, Henstedt-Ulzburg, Germany; 3 Research Center Borstel, Medical Clinic, Parkallee 35, D-23845 Borstel, Germany

## Abstract

**Background:**

As there is no optimal treatment of non small cell lung cancer due to its resistance to common chemotherapeutics, we investigated the effect of human placenta-conditioned medium on tumor tissue. The human placenta constitutes a mixture of maternal and fetal origin and displays a variety of immunomodulatory aspects.

**Methods:**

Freshly resected non small cell lung cancer tissues were incubated with placenta-conditioned medium in a short-term tissue culture model and A549 cells were challenged, respectively. Term placenta was used for producing conditioned medium and HOPE-fixed stimulated tumor tissue was analyzed for expression of caspase-3 and Ki67 via immunohistochemistry. The effects of conditioned medium on squamous cell carcinoma were further compared to physiological concentrations of Carboplat/Gemzar.

**Results:**

Conditioned medium caused in 2 of 3 cases elevated expression of caspase-3 and reduced expression of Ki67 in 3 out of 3 cases, while the chemotherapeutic agents caused no comparable expression of caspase-3 or reduction of Ki67. In cell culture up to 50% of karyopyknosis was investigated and even sterile-filtrated medium caused widespread reduction of Ki67 on protein level.

**Conclusion:**

Human placenta releases substances that mediate apoptosis and reduce proliferation in tumor tissue and cell culture. As even sterile-filtrated medium caused the mentioned effects we hypothesize one or more soluble mediators. The detailed way of promoting apoptosis and nature of these mediators need to be elucidated in further studies.

## Findings

Although new treatment regimens are currently being developed, Non small cell lung cancer (NSCLC) still remains a fatal diagnosis and the success in its treatment up to date is not satisfying. Due to the poor response to common chemotherapeutics there are multifaceted endeavors to resolve these obstacles. This study focused on the effects of placenta-conditioned medium on human lung carcinoma.

### The placenta has immunomodulatory properties

The human placenta as a unique structure of maternal and fetal origin has become a focus of interest in recent publications. The semi allograft fetus is a challenge for the maternal immune system and the exact way of avoiding its rejection is up to date not completely known. The implanting blastocyst adheres to the uterine wall and soon thereafter cytotrophoblast cells invade the endometrium to establish in cooperation with the maternal tissue a suitable habitat for the growing fetus. The processes after implantation resemble in many aspects some traits of neoplastic cells. As these cells have to gain access to the bloodstream via *de novo *vascularization, produce their own growth factors and invade adjacent tissue by the use of proteases like cathepsins or matrix-metalloproteinases (MMPs), one can speak of a transient pseudo-tumorigenesis [1]. As a stranger in a strange land, the fetus has to evade and mediate its mother's immune response through many sophisticated mechanisms as reviewed elsewhere; not only alone but rather in a dialogue with the temporary host [2]. A remarkable micro array study of placental expression patterns revealed a rich diversity of tissue and function restrictions [3] and overexpression of placental growth factor (PlGF) in cell culture seemed to inhibit tumor growth in a mouse model [4].

### Growth suppression of transformed cells through placental extract

As evidence of a possible effect of gene transcription and transformed cell growth suppression by placental extract is not new [5-7] we examined in this study for the first time the effect of placenta-conditioned medium on human tumor tissue and in cell culture, respectively. To test whether placental-conditioned medium has an effect on apoptosis and proliferation the expression of effector caspase-3 and of Ki67 were analyzed with immunohistochemistry in NSCLC-tissues and A549 cells. Sterile filtrated and non-filtrated conditioned medium caused expression of caspase-3 and a reduced expression of Ki67 in comparison to medium controls. Term placental tissue was obtained from healthy patients after cesarean section and immediately processed for tissue and cell culture in the following manner: For conditioned medium the placental tissue was cut into small pieces approx. 0,6 cm^3^, transferred into 6 ml RPMI-1640 (Gibco^®^, Invitrogen, Karlsruhe) and incubated for not more than 4 h at 37°C. For sterile filtration of media a Rotilabo^®^-syringe-filter (Carl Roth GmbH, Karlsruhe) with 0.22 μm cut-off size was used. Filtrated and unfiltrated media were used for tissue culture and HOPE-fixation according to Lang *et al*. [8] with freshly resected tumor tissue. As negative control, tumor tissue was treated with RPMI-1640. After fixation with HOPE-fixative the paraffin-embedded tissue was deparaffinized and rehydrated as previously described [9]. The primary antibody (anti-Caspase-3-cleaved, clone: CI752C002, DCS Innovative Diagnostik Systeme, Hamburg and anti-Ki67, clone: MIB-1, Dako, Hamburg) was incubated for 30 min. in a 1:100 dilution. For sensitive detection with permanent AEC (Zytomed Systems) as chromogen an enzyme-polymersystem (ZytochemPlus HRP Polymer Kit, Zytomed Systems) was used. Tissue sections were counterstained by incubation in Mayer's haemalum for 5 minutes.

### Upregulation of Caspase-3 and suppression of Ki67 in squamous cell carcinoma by placenta-conditioned medium

**Figure 1:**
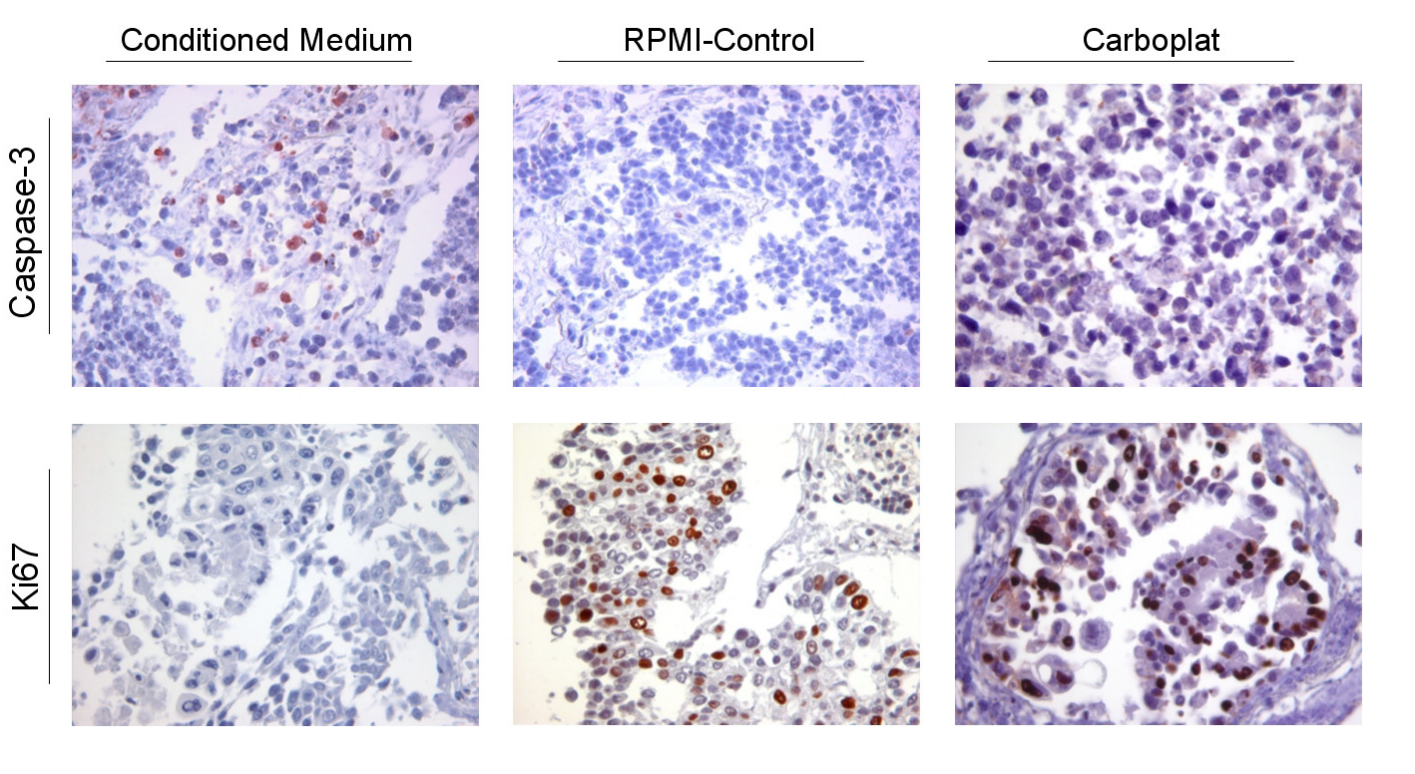
**Immunohistochemical detection of Caspase-3 (upper panel) and Ki67 (bottom panel)**. Squamous cell carcinoma tissue was incubated with placenta-conditioned medium (left column) and with RPMI (middle column) or Carboplat (right column).

Immunohistochemistry staining detected an elevated expression of caspase-3 (N = 2) and reduced expression of Ki67 (N = 3) after incubation of 16 h with conditioned medium (Figure 1). This indicates an apoptotic-promoting and growth-restricting effect of the conditioned medium. Whether this might be due to death-inducing TNF-superfamily ligands, which are abundant in placenta [10] remains to be elucidated. Common chemotherapeutic agents like Carboplat/Gemzar, which were applied in a typical therapy dose rate like previously described [8], caused no induction of Caspase-3 expression and a comparably small effect on proliferation; exemplified for Carboplat in Figure 1. To exclude reactions between tumor tissues and remaining immune cells from the placental tissues, the effects of conditioned media on A459 cells were further analyzed.

### Apoptosis and reduced proliferation of A549 cells

**Figure 2:**
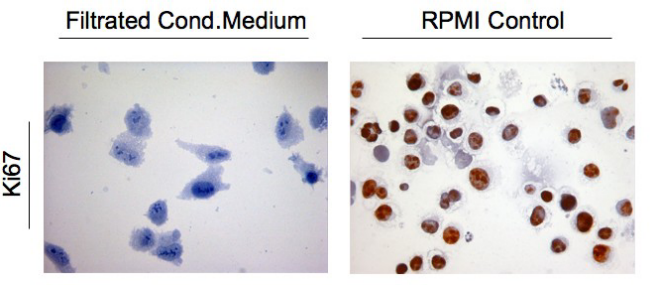
**Immunohistochemical detection of Ki67 on cytospin fixed A549 cells after incubation with sterile-filtrated placenta-conditioned medium**.

**Figure 3:**
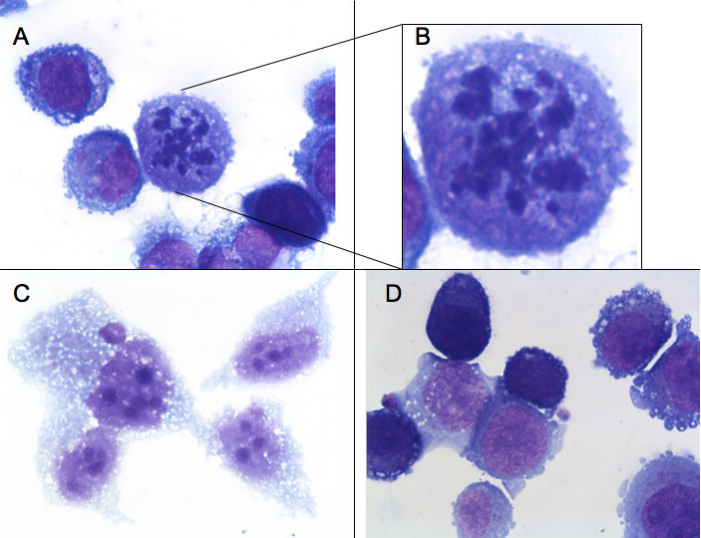
**A549 cell culture with sterile-filtrated placenta-conditioned medium (A, B and C) or RPMI (D)**.

A549 cells were treated equally as lung tumor tissue and thereafter fixed via cytospin preparation on cover slides. Even sterile filtrated conditioned medium, which doesn't contain any immunological active cells like Hofbauer cells, caused constantly reduced expression of Ki67 (Figure 2) and karyopyknosis (Figure 3). This supports the results from tissue culture experiments. To draw a conclusion, there is evidence to suggest that placenta-conditioned medium contains ingredients which promote apoptosis and reduce proliferation in human tumor tissues. Even as the filtrated medium promotes the mentioned effects we hypothesize that some sort of soluble factors are the conducting elements and we recommend further studies about their nature. We are well aware that the amount of samples analyzed here demands validation in further studies. The reason for the limited sample size is the challenge of well-timed logistics to simultaneously obtain fresh placenta and tumor tissues. Implementation of automatic sample analysis techniques [11,12] could reduce subjective influences in the above mentioned case and refine the drafted conclusions.

Nevertheless, we clearly showed antitumoral effects of substances which are released from placenta in human NSCLC tissues and A549 cells. The antitumoral effects from placenta were stronger than those obtained with conventional chemotherapeutics in the same model. Further studies are underway to characterize the active substances released by the placenta. We conclude that the pro-apoptotic and anti-proliferative properties of substances released by the human placenta to NSCLC may lead to novel treatment approaches.

## Abbreviations

(NSCLC): Non small cell lung cancer; (MMPs): Matrix-metalloproteinases; (PlGF): Placental growth factor; (STST): Short term stimulation of tissues; (HOPE): Hepes Glutamic Acid Buffer Mediated Organic Solvent Protection Effect; (TNF): Tumor Necrosis Factor.

## Competing interests

The authors declare that they have no competing interests.

## Authors' contributions

TG conceived of the study. SM conducted the experimental work, analyzed the sections and drafted the document with TG. TZ, HS and EV were involved in surgical and pathological parts, respectively. DK and MA took part in immunohistochemistry. HPH and PZ were involved in cell culture and pneumological aspects. All authors have read and approved the present manuscript.

## References

[B1] Hunt JS (2006). Stranger in a strange land. ImmunolRev.

[B2] Soundararajan R, Jagannadha Rao A (2004). Trophoblast 'pseudo-tumorigenesis': Significance and contributory factors. Reproductive Biology and Endocrinology.

[B3] Sood R, Zehnder JL, Druzin ML, Brown PO (2006). Gene expression patterns in human placenta. Proc Natl Acad Sci USA.

[B4] Xu L, Cochran DM, Tong RT, Winkler F, Kashiwagi S, Jain RK, Fukumura D (2006). Placenta growth factor overexpression inhibits tumor growth, angiogenesis, and metastasis by depleting vascular endothelial growth factor homodimers in orthotopic mouse models. Cancer Res.

[B5] Zhu JH, Pang ZJ, Chen SL, Xing FQ (2004). Effect of decidual cell conditioned media on invasion-related gene expression of ovarian tumor cell line COC1. Di Yi Jun Yi Da Xue Xue Bao.

[B6] Klein JL, Hamel E, Tayot JL, Yamasaki H (1991). Growth suppression of transformed cells by a human placental extract not related to ransforming growth factor β*. J Cancer Res Clin Oncol.

[B7] Bischof P, Meisser A, Campana A, Tsend L (1998). Effects of decidua-conditioned medium and insulin-like growth factor binding protein-1 on trophoblastic matrix metalloproteinases and their inhibitors. Placenta.

[B8] Lang DS, Droemann D, Schultz H, Branscheid D, Martin C, Ressmeyer AR, Zabel P, Vollmer E, Goldmann T (2007). A novel human ex vivo model for the analysis of molecular events during lung cancer chemotherapy. Respir Res.

[B9] Schultz H, Kähler D, Branscheid D, Vollmer E, Zabel P, Goldmann T (2008). TKTL1 is overexpressed in a large portion of non-small cell lung cancer specimens. Diagn Pathol.

[B10] Philips TA, Ni J, Hunt JS (2001). Death-inducing tumor necrosis factor (TNF) superfamily ligands and receptors are transcribed in human placentae, cytotrophoblasts, placental macrophages and placental cell lines. Placenta.

[B11] Kayser G, Radziszowski D, Bzdyl P, Sommer R, Kayser K (2006). Theory and implementation of an electronic, automated measurement system for images obtained from immunohistochemically stained slides. Anal Quant Cytol Histol.

[B12] Pham NA, Morrison A, Schwock J, Aviel-Ronen S, Iakovlev V, Tsao MS, Ho J, Hedley DW (2007). Quantitative image analysis of immunohistochemical stains using a CMYK color model. Diagnostic Pathology.

